# Graph —Hoof Disease in the Horse

**Published:** 1881-05

**Authors:** R. R. Gregg

**Affiliations:** Buffalo, N. Y.


### DISEASE OF THE HOOF IN A HORSE.

BY R. R. GREGG, M.D., BUFFALO, N. Y.

In April 1865 one of my horses was disabled by a "quarter crack" on the inside of left forefoot. The crack extended from the hair down through the whole hoof, and finally opened to the extent of about a quarter of an inch in the widest place. The horse was entirely disabled for use. The two parts of the hoof worked upon each other to such an extent as to cut into the "quick" and fleshy parts at the top, so that after being driven but a few blocks, there would be bleeding from the crack sufficient to leave a pool of blood when the horse was left standing a few minutes. I engaged a veterinary surgeon, not feeling able to select the homœopathic remedy for such a case. He carefully pared the edges of the crack, and injected stimulating lotions; subsequently hot tar was poured into the crack; and finally other means were resorted to; the horse however, continued to get worse. I then sought other advice, but was told that there was no cure except putting the horse out to pasture for the entire summer, and it was doubtful if the hoof would ever become thoroughly sound.

I now resolved to try homœopathic treatment. During the two or three months in which the hoof had been getting into this condition, all the hoofs became drier than natural and threw out ridges more or less, especially the cracked one, which had also become quite seriously contracted all around below the hair. Following the indications of cracked, dry, and ridged nails in man, I selected *Graphites*, and gave one dose (about a dozen pellets) of the 6000th potency, dry on the tongue. The next day there was a perceptible improvement in the lameness; the second day it was much better; and on the third day I commenced driving the horse cautiously; in a few days all lameness disappeared. In three weeks he began to limp again, and I then repeated the dose. The lameness disappeared again in a day or two, and did not return, even under very hard driving, for six weeks. Then there was a slight return, and I gave one more dose. *From this time the horse was never lame for the eleven years during which I continued to drive him, even with the hardest usage.*

As the growing part of the cracked hoof extended down below the hair, I found it firmly united in the line of the crack, and it grew off in this way, being entirely closed up as soon as the old half was all grown off, and so it ever afterward continued. The contracted and ridged appearance also disappeared. It was a cold wet spring when the lameness developed, but so soon as he was sufficiently well, I used him in all weather, frequently driving him through rain and mud all day; the crack of course filled with water and mud under these circumstances, but this did not appear to retard the cure.

The same horse had, in the fall of 1873, a severe attack of the epizootic which then prevailed so universally over this country. I treated him homœopathically through the first stage without unusual symptoms. In the second stage, the purulent discharge from the nostrils became the most excessive of any among a large number of horses where he was kept; the manger and sides of the stalls, as far back as he was able to reach his head, were completely and continuously besmeared with the thick yellow and tenacious discharge which he was almost constantly trying to wipe from his nose. After giving a few remedies and then waiting for several days, the horse getting worse in the mean time, I gave one dose of *Pulsatilla* 1000, which almost wholly subdued the yellow discharge by the next day, and entirely so in another twenty-four hours, leaving only a little watery discharge for a day or two longer, with a restoration to the soundest health in a week or less from the administration of that one dose.

